# Healthcare services efficiency and its intrinsic drivers in China: based on the three-stage super-efficiency SBM model

**DOI:** 10.1186/s12913-023-09820-x

**Published:** 2023-07-29

**Authors:** Mengya Sun, Yaojun Ye, Guangdi Zhang, Xiuling Shang, Yuan Xue

**Affiliations:** 1 College of Science, Zhejiang University of Science and Technology, Hangzhou, China; 2 The Third Department of Critical Care Medicine, Shengli Clinical Medical College of Fujian Medical University, Fujian Provincial Hospital, Fujian Provincial Center for Critical Care Medicine, Fujian Provincial Key Laboratory of Critical Care Medicine, Fuzhou, China; 3 Operation and Management Office, Fujian Provincial Hospital, Fuzhou, China

**Keywords:** Healthcare services efficiency, Healthcare services efficiency growth, Super-efficient SBM model, GML index

## Abstract

**Background:**

The purpose of this study is to examine the development of healthcare services efficiency in China since the reform of the healthcare system. By examining the development environment of healthcare services in China and examining the driving factors affecting the efficiency of healthcare services, we provide a reference for the future high-quality development of healthcare services in China.

**Methods:**

A three-stage super-efficient slack-based measure (SBM) model with undesirable outputs was used to measure the efficiency of healthcae services in 31 Chinese provinces from 2009 to 2021, and a global Malmquist-Luenberger (GML) index was used to assess their spatiotemporal evolution characteristics and internal influencing mechanisms of healthcare services efficiency.

**Results:**

The empirical results showed that the efficiency of China's healthcare services changed significantly from 2009–2014 and then remained stable. During the study period, the efficiency of healthcare services in the eastern region was higher than the national level, while it was lower in the western region. The results of the analysis of environmental factors indicated that an increase in population density reduced the redundancy of healthcare input resources and that economic development as well as an increase in government subsidies, contributed to an increase in the redundancy of healthcare input resources. The main contribution to the growth of healthcare sercices efficiency in China came from the technological innovation effect, and the growth was most significant in the western region.

**Conclusion:**

From 2009 to 2021, the efficiency of national healthcare services generally showed a slow upward trend, and the efficiency of healthcare services varied widely among regions. Under the existing environmental constraints, relevant departments in each region should strengthen technological innovation in healthcare services, completely focus on the regional catch-up effect, and promote the balanced development of regional health.

## Background

In March 2009, the introduction of the Opinions on Deepening the Reform of the Medical and Health System marked the official launch of the “healthcare reform” in China, intending to provide universal coverage of basic medical services to all Chinese citizens by 2020 [1]. Over the past decade, China's investment in medical resources has gradually increased, and health conditions have been gradually improving. In 2021, the national government health expenditure reached 206,760,600 million yuan, the total number of medical and health institutions reached 10,309,035, and the number of medical personnel reached 13,985,400 showing an increase of 329.30%, 12.48% and 79.73%, respectively, compared with 2009. In terms of “medical output", the number of consultations reached 8.472 billion, and the number of hospital discharges reached 246.421 million in 2021, with an increase of 54.38% and 85.11%, respectively, compared to 2009. These data show that China's medical reform has achieved certain results. However, the problem of "difficult and expensive medical care" is becoming increasingly prominent, and the unbalanced allocation of medical resources and unreasonable structural layout make it difficult for China's healthcare system to prevent and control major diseases and respond to public health emergencies. China's medical reform is still a long way to go. These challenges in China's healthcare system require government departments to find new solutions. Increasing public healthcare spending on a large scale to achieve equalization of healthcare services is still not practical, and the focus should shift to improving the efficiency of healthcare services [2]. The Implementation Plan for Deepening the Reform of the Medical and Health System 2009–2011 clearly states that the efficiency of healthcare services should be scientifically evaluated and that the efficiency of healthcare services is a key indicator of the effectiveness of healthcare reform. A proper assessment of the efficiency of healthcare services is a prerequisite for improving the quality of healthcare services. This study attempts to evaluate the service status of Chinese healthcare institutions and explore the factors inherent in the growth of healthcare services efficiency. Identify the problems of China's healthcare services, and then provide a basis for the rational allocation of healthcare resources and the coordinated development of regional health in China.

Since 1917, when a professional quality appraisal organization was first established in the United States to evaluate the efficiency of healthcare organizations, the evaluation of healthcare services efficiency and the investigation of its influencing factors have gradually become a research hotspot. The data envelopment analysis (DEA) method is currently the most used for assessing healthcare services efficiency. Mazon et al. [3] used data envelopment analysis to assess the technical efficiency of municipalities in Santa Catarina in terms of public health spending and explored its relationship to regulatory conditions. The efficiency of healt systems in OECD countries was assessed using network data envelopment analysis (DNDEA) to inform the development of strategic health plans in OECD countries [4]. Lobo et al. developed a dynamic data envelopment analysis assessment tool to evaluate the efficiency of the Federal University General Hospital [5]. The results of Bağci and Konca's study of tertiary care hospitals suggest that hospital size or university hospital status affects the technical efficiency of hospitals [6].

In response to the current problems of unequal distribution of resources and uncoordinated regional development faced by Chinese healthcare institutions, many scholars have conducted studies. To examine the imbalance in the distribution of regional medical resources in China, Sun et al. combined weighted data envelopment analysis and game theory to establish a healthcare services efficiency evaluation model, concluding that coastal regions have the highest healthcare services efficiency [7]. Zhou’s study of primary health institutions in Jiangsu Province showed that deepening healthcare reform has led to significant productivity growth in community health centers, but the efficiency of healthcare services is deteriorating [8]. Li et al. combined a DEA model with a qualitative comparison of fuzzy sets to analyze the determinants affecting technical efficiency, and the results show that the path to achieve high technical efficiency consists of high mortality and high fiscal allocation, high population density and high GDP dominance [9]. However, traditional DEA models do not consider input and output slack when assessing healthcare services efficiency and may lead to biased calculations due to radial and angular choices. To overcome this problem, Tone [10] proposed a non-radial, non-angular SBM model. However, the efficiency values of the effective decision-making units of this method are all 1, and it is not possible to compare the ranking of multiple effective decision-making units. On the other hand, the super-efficiency SBM model allows efficiency values greater than 1 and has been widely used in recent years for total factor productivity (TFP) [11, 12] and eco-efficiency measurement [13],yet, this approach has been rarely applied to the assessment of healthcare services efficiency. In addition, any input–output process will obtain desirable and undesirable outputs, and healthcare services are certainly no exception. Furthermore, for the study object of panel data, the index calculated using an adjacent frontier benchmark may have the problem of no feasible solution [14]. To avoid the errors caused by the methodological limitations of traditional DEA, some scholars have combined DEA models with other methods. For example, Top et al. [15] and Sun et al. [16] measured the efficiency of healthcare systems through a DEA-Tobit two-stage model. However, the Tobit model only considers the effects of environmental factors and does not identify the effects of statistical noise on different decision-making units.

In view of the limitations of the current study, we introduce a super-efficient SBM model that includes non-desired outputs to assess the services efficiency of healthcare institutions. In addition, we propose an efficiency index calculation method based on the global benchmarking technique, which makes the results of the global benchmarking technique comparable in horizontal space and vertical time compared with the adjacent benchmarking technique, enhancing the scientific validity of cross-year and cross-province comparisons. In addition, in order to exclude the influence of environmental factors and random noise on the efficiency measures of health institutions' medical services, we borrow SFA regression to introduce environmental factors and random noise into the model simultaneously.

The main innovations of this study are the following: first, considering the possible problem of no feasible solution or non-transferability using adjacent frontier benchmarking techniques, we used global benchmarking techniques for measuring healthcare services efficiency. A new frontier was constructed as a unified measurement benchmark by considering the same samples from different observation periods as different DMUs and mixing them into a set. Second, overcoming the limitations of traditional DEA, this paper innovatively combined the super-efficiency SBM model based on slack measure and stochastic frontier analysis (SFA) method and added the consideration of undesirable outputs in an attempt to obtain a more objective and realistic picture of healthcare service efficiency. Third, this study not only calculated the growth of healthcare services eficiency in 31 Chinese provinces but also examined the intrinsic drivers of healthcare services efficiency growth. Therefore, we measured and analyzed China’s healthcare services efficiecy (HSE), healthcare services efficiency growth index (HSGI), and its two decomposition terms. Finally, the characteristics of healthcare service efficiency growth were studied, and their intrinsic drivers were explored.

## Methods

### Methodology

The three-Stage Global-SBM super-efficiency DEA model was constructed by combining the super-efficiency SBM model, which includes non-desirable outputs and the SFA model that circumvent non-management factors while considering the global benchmarking technique. The model is also used to measure and analyze the healthcare services efficiency, healthcare services efficiency growth index, and their decomposition in 31 provinces of China.

#### Three-Stage Global-SBM super-efficiency DEA model with Undesirable outputs

To effectively solve the problems caused by the traditional DEA model due to radial and angular, Tone and his team improved the traditional SBM model and proposed the SBM model considering undesired outputs and the super-efficient SBM model; the former introduced the undesirable output into the model, and the latter well solved the ranking problem among multiple effective decision-making units. Based on this, to more accurately measure the efficiency of healthcare services in China, this study referred to the method of Li and Shi [17], i.e., it combined the super-efficient SBM model and the undesirable SBM model and constructed the undesirable output-super-efficient SBM model. Considering that healthcare services may be influenced by management and non-management factors, this paper adopted the SFA method to circumvent the interference brought by non-management factors and obtain more realistic efficiency values. The specific steps were as follows.

- **Stage 1:** The Global-SBM super-efficiency DEA model.

A production system with $n$ DMUs was considered; each DMU has $m$ inputs, $q_{1}$ desirable outputs, and $q_{2}$ kinds of undesirable outputs. The input and output variables for each DMU are $X = (x_{1} ,x_{2} ,x_{3} , \cdots ,x_{m} ) \in R_{m}^{ + }$,$Y = (y_{1} ,y_{2} ,y_{3} , \cdots ,y_{{q_{1} }} ) \in R_{{q_{1} }}^{ + }$, and $B = (b_{1} ,b_{2} ,b_{3} , \cdots ,b_{{q_{2} }} ) \in R_{{q_{2} }}^{ + }$, where $X > 0,Y > 0,B > 0$ are assumed. The production possibility set (P) is defined as

$$P = \left\{ {(x,y,b)|x \ge X\lambda ,y \le Y\lambda ,b = B\lambda ,\lambda \ge 0} \right\}$$

where $\lambda$ is the intensity vector. According to the SBM model of Tone [18] and the assumption of weak disposability, the SBM model used to evaluate the undesirable processing output of the DMU is defined as follows.

1 $$\begin{array}{*{20}l} \rho = \min \frac{{1 - \frac{1}{m}\sum\nolimits_{i = 1}^{m} {\frac{{s_{i}^{ - } }}{{x_{i0} }}} }}{{1 + \frac{1}{{q_{1} + q_{2} }}(\sum\nolimits_{k = 1}^{{q_{1} }} {\frac{{s_{k}^{ + } }}{{y_{k0} }} + \sum\nolimits_{l = 1}^{{q_{2} }} {\frac{{s_{l}^{b - } }}{{b_{l0} }})} } }} \hfill \\ \\ s.t.\left\{ \begin{aligned} & x_{i0} = \sum\nolimits_{j = 1}^{n} {\lambda_{j} x_{j} + s_{i}^{ - } } ,\forall i \hfill \\ & y_{k0} = \sum\nolimits_{j = 1}^{n} {\lambda_{j} y_{j} - s_{k}^{ + } } ,\forall k \hfill \\ & b_{l0} = \sum\nolimits_{j = 1}^{n} {\lambda_{j} b_{j} + s_{l}^{b - } } ,\forall l \hfill \\ & \sum\nolimits_{j = 1}^{n} {\lambda_{j} = 1} \hfill \\ & s_{i}^{ - } \ge 0,s_{k}^{ + } \ge 0,s_{l}^{ - } \ge 0,\lambda_{j} \ge 0,\forall i,j,k,l \hfill \\ & i = 1,2, \cdots ,m;k = 1,2, \cdots ,q_{1} ;l = 1,2, \cdots ,q_{2} \hfill \\ \end{aligned} \right. \hfill \\ \end{array}$$

where $s_{i}^{ - } ,s_{k}^{ + } ,s_{l}^{b - }$ correspond to the slack of the input, desirable output, and undesirable output, respectively. The function value of the optimization $\rho$ is the efficiency value of the making decision unit $(x_{0} ,y_{0} ,b_{0} )$.

However, the empirical results often have multiple decision-making units in the “efficient state”. Therefore, reasonably distinguishing these efficient decision-making units is important for efficiency ranking and influence factor analysis. Based on the above non-expectation SBM model, we constructed a super-efficient SBM model with non-expectation output to evaluate the efficient DMUs.

2 $$\begin{array}{*{20}l} {\rho^{*} = \min \frac{{1 + \frac{1}{m}\sum\nolimits_{i = 1}^{m} {\frac{{s_{i}^{ - } }}{{x_{i0} }}} }}{{1 - \frac{1}{{q_{1} + q_{2} }}(\sum\nolimits_{k = 1}^{{q_{1} }} {\frac{{s_{k}^{ + } }}{{y_{k0} }} + \sum\nolimits_{l = 1}^{{q_{2} }} {\frac{{s_{l}^{b - } }}{{b_{l0} }})} } }}} \\ \\ {s.t.\left\{ \begin{aligned} & x_{i0} \ge \sum\nolimits_{j = 1,j \ne 0}^{n} {\lambda_{j} x_{j} - s_{i}^{ - } } ,\forall i \hfill \\ & y_{k0} \le \sum\nolimits_{j = 1,j \ne 0}^{n} {\lambda_{j} y_{j} + s_{k}^{ + } } ,\forall k \hfill \\ & b_{l0} \ge \sum\nolimits_{j = 1,j \ne 0}^{n} {\lambda_{j} b_{j} - s_{l}^{b - } } ,\forall l \hfill \\ & 1 - \frac{1}{{q_{1} + q_{2} }}(\sum\nolimits_{k = 1}^{{q_{1} }} {\frac{{s_{k}^{ + } }}{{y_{k0} }} + \sum\nolimits_{l = 1}^{{q_{2} }} {\frac{{s_{l}^{b - } }}{{b_{l0} }})} } > 0 \hfill \\ & \sum\nolimits_{j = 1}^{n} {\lambda_{j} = 1} \hfill \\ & s_{i}^{ - } \ge 0,s_{k}^{ + } \ge 0,s_{l}^{ - } \ge 0,\lambda_{j} \ge 0,\forall i,j,k,l \hfill \\ & i = 1,2, \cdots ,m;k = 1,2, \cdots ,q_{1} ;l = 1,2, \cdots ,q_{2} \hfill \end{aligned} \right.}\end{array}$$

where $\rho^{*}$ is the calculated global comprehensive technical efficiency (TE^G^) of healthcare services expressed by HSE. Note that the above model is performed under the assumption of constant returns to scale (CRS). By adding the restriction $\sum\nolimits_{j = 1}^{n} {\lambda_{j} = 1}$ to $\lambda$ on this basis, it becomes a model under the assumption of variable returns to scale (VRS), and the results obtained at this point are called global pure technical efficiency (PTE^G^). The comprehensive technical efficiency can be further decomposed into pure global technical efficiency and global scale efficiency (SE^G^). HSE is calculated using the following formula: $HSE = \, PTE^{G} \times SE^{G}$.

- **Stage 2:** The Stochastic Frontier Analysis (SFA) model and variable input adjustment.

Fried et al. [19] argued that managerial inefficiency, environmental effects, and statistical noise affect the slack variables reflecting the inefficiency of the decision units. Therefore, the second stage aimed to remove the effects of environmental factors and statistical noise from the slack variables obtained in the first stage and to adjust all decision units in the same external environment. To achieve this goal, which could be done only with the help of SFA regression, the following steps were applied:

First, SFA regression analysis is performed on the slack values of the input variables obtained in the first stage, using the environmental variables and the mixed error term as explanatory variables, where the mixed error term includes managerial inefficiency and statistical noise. The formula is as follows:

3 $$S_{ni} = f(Z{}_{i};\beta_{n} ) + \nu_{ni} + \mu_{ni} ;i = 1,2, \cdots ,I;n = 1,2, \cdots ,N$$

where $S_{ni}$ is the slack value of the nth input of the ith decision unit; $Z{}_{i}$ is the environmental variable, and $\beta_{n}$ is the coefficient of the environmental variable; $\nu_{ni} + \mu_{ni}$ is the mixed error term, denoted by $\varepsilon$, where $\nu_{ni}$ signifies statistical noise and $\mu_{ni}$ means management inefficiency.$\nu \sim N(0,\sigma_{v}^{2} )$ denotes the effect of random disturbances on the input slack variables;$\mu$ denotes the effect of management factors on the input slack variables, which is assumed to obey a normal distribution truncated at zero, i.e.,$\mu \sim N^{ + } (0,\sigma_{\mu }^{2} )$.

The purpose of the SFA regression is to remove the effects of environmental factors and statistical noise on efficiency in order to adjust all decision-making units to the same external environment. The following discussion aims to separate out environmental factors and statistical noise.

In this study, the formula for separating management inefficiency is derived with reference to Jondrow's idea in the following form:

$$E\left( {\mu \left| \varepsilon \right.} \right){ = }\sigma_{*} \left[ {\frac{{\varphi (\lambda \frac{\varepsilon }{\sigma })}}{{\Phi (\frac{\lambda \varepsilon }{\sigma })}} + \frac{\lambda \varepsilon }{\sigma }} \right],E[\nu_{ni} |\nu_{ni} + \mu_{ni} ] = S_{ni} - f(Z{}_{i};\beta_{n} ) - E[\mu_{ni} |\nu_{ni} + \mu_{ni} ]$$

where, $$\sigma_{*} = \frac{{\sigma_{\mu } \sigma_{\nu } }}{\sigma },\sigma = \sqrt {\sigma_{\mu }^{2} + \sigma_{\nu }^{2} }$$,$$\lambda { = }\sigma_{\mu } {/}\sigma_{\nu }$$。

In the specific calculation, $\sigma^{2} ,\gamma$ are derived from the SFA regression results, $\sigma_{v}$ and $\sigma_{\mu }$ can be obtained by $\sigma^{2} = \sigma_{v}^{2} + \sigma_{\mu }^{2}$ and $\gamma = \frac{{\sigma_{\mu }^{2} }}{{\sigma_{v}^{2} + \sigma_{\mu }^{2} }}$. If $\gamma$ converges to 1, it indicates that managerial inefficiency is the dominant factor in efficiency perturbation.

Finally, the original inputs were adjusted by the SFA regression results to achieve error correction with the following adjustment formula:$X_{ni}^{A} = X_{ni} + [\max (f(Z_{i} ;\mathop \beta \limits^{ \wedge }_{n} )) - f(Z_{i} ;\mathop \beta \limits^{ \wedge }_{n} )] + [\max (\nu_{ni} ) - \nu_{ni} ] \, i = 1,2, \cdots ,I;n = 1,2, \cdots ,N$ where $X_{ni}^{A}$ is the adjusted input; $X_{ni}$ is the pre-adjusted input;$[\max (f(Z_{i} ;\mathop \beta \limits^{ \wedge }_{n} )) - f(Z_{i} ;\mathop \beta \limits^{ \wedge }_{n} )]$ is an adjustment for external environmental factors and $[\max (\nu_{ni} ) - \nu_{ni} ]$ is placing all decision-making units at the same level of luck.

- **Stage 3:** Adjusted SBM‑DEA‑BBC model.

The input variables corrected by the SFA regression model and raw output data are substituted into the SBM-BBC model for calculation, and the efficiency at this point is relatively realistic and accurate as the effects of environmental factors and statistical noise have been removed.

#### Global-Malmquist-Luenberger (GML) index and its decomposition

To analyze the intrinsic drivers of productivity change in DMUs, it is necessary to use the Malmquist-Luenberger (ML) index. Färe et al. [20] first calculated the M-index using the DEA method and decomposed the M-index into technical efficiency changes (TE) and technical changes (TC). Chung et al. [21] applied the directional distance function containing undesirable outputs to the M-index model, called the ML index. Subsequently, scholars began to refer to the ML index as containing undesirable outputs. Pastor and Lovell [22] constructed the GM index and decomposed it into technical efficiency change (EC) and technical gap change (BPC). In this paper, we referred to Paster's construction method for the GM index and constructed the global-Malmquist-Luenberger (GML) index, whose calculated value represents the growth in efficiency of healthcare services expressed in terms of HSGI.

A panel consisting of decision-making units $i = 1,2, \ldots ,I$ and time periods $t = 1, \ldots ,T$ was considered. The producer uses input $x \in R_{m}^{ + }$ to produce output $y \in R_{q}^{ + }$. Herein, we defined two techniques, and the contemporaneous benchmark technique was defined as $T_{c}^{t} = \left\{ {(x^{t} ,y^{t} )|x^{t} can \, produce \, y^{t} } \right\}$, in which $\lambda T_{c}^{t} = T_{c}^{t} ,t = 1, \cdots ,T,\lambda > 0$. The global benchmarking technique is defined as $T_{c}^{G} = conv \, \left\{ {T_{c}^{1} \cup \cdots \cup T_{c}^{T} } \right\}$. Since the evaluated DMUs are all included in the global reference technology set, the GML index based on the global DEA model did not have the problem of no feasible solution for the VRS (variable payoff for scale) model. Given the above advantages of the GML index, both benchmarking techniques in this paper were calculated under the condition of constant payoff to scale.

The global Malmquist-Luenberger productivity index based on the same period $T_{c}^{G}$ was defined as

4 $$HSGI_{c}^{G} (x^{t} ,y^{t} ,z^{t} ,x^{t + 1} ,y^{t + 1} ,z^{t + 1} ) = \frac{{D_{c}^{G} (x^{t + 1} ,y^{t + 1} ,z^{t + 1} )}}{{D_{c}^{G} (x^{t} ,y^{t} ,z^{t} )}}$$

where, $D_{c}^{G} (x,y,z) = \min \left\{ {\phi > 0|(x,y,z|\phi ) \in T_{c}^{G} } \right\}$ was the output distance function.

$HSGI_{c}^{G}$ could be decomposed into

5 $$\begin{gathered} HSGI_{c}^{G} (x^{t} ,y^{t} ,z^{t} ) = \frac{{D_{c}^{{\text{t + 1}}} (x^{t + 1} ,y^{t + 1} ,z^{t + 1} )}}{{D_{c}^{t} (x^{t} ,y^{t} ,z^{t} )}} \times \left\{ {\frac{{D_{c}^{{\text{G}}} (x^{t + 1} ,y^{t + 1} ,z^{t + 1} )}}{{D_{c}^{t + 1} (x^{t + 1} ,y^{t + 1} ,z^{t + 1} )}} \times \frac{{D_{c}^{{\text{t}}} (x^{t} ,y^{t} ,z^{t} )}}{{D_{c}^{G} (x^{t} ,y^{t} ,z^{t} )}}} \right\} \hfill \\ \, = \frac{{TE_{c}^{{\text{t + 1}}} (x^{t + 1} ,y^{t + 1} ,z^{t + 1} )}}{{TE_{c}^{t} (x^{t} ,y^{t} ,z^{t} )}} \times \left\{ {\frac{{D_{c}^{{\text{G}}} (x^{t + 1} ,y^{t + 1} ,z^{t + 1} )/D_{c}^{t + 1} (x^{t + 1} ,y^{t + 1} ,z^{t + 1} )}}{{D_{c}^{G} (x^{t} ,y^{t} ,z^{t} )/D_{c}^{{\text{t}}} (x^{t} ,y^{t} ,z^{t} )}}} \right\} \hfill \\ \, = EC_{{\text{c}}} \times \left\{ {\frac{{BPG_{c}^{G,t + 1} (x^{t + 1} ,y^{t + 1} ,z^{t + 1} )}}{{BPG_{c}^{G,t} (x^{t} ,y^{t} ,z^{t} )}}} \right\} = EC_{{\text{c}}} \times BPC_{c} \hfill \\ \end{gathered}$$

where $EC$ represents the change in healthcare services efficiency of $DMUs$ from period $t$ to $t + 1$ and describes the “catch-up effect” in the current benchmark technology frontier. EC > 1 (or < 1) indicates that the DMU is closer to (or farther from) the current benchmark technology frontier than in the previous period.$BPG$(Best Practice Gap) is the gap between the current benchmark technology frontier and the global benchmark frontier. BPC (Best Practice Change) is the change of $BPG$ from period $t$ to $t + 1$.$BPC$ reflects the change in the healthcare services efficiency gap between the global benchmark technology and the current benchmark technology and describes the “innovation effect” of the current healthcare services.$BPC$ > 1 (or < 1) indicates that the current benchmark technology frontier is closer to (or farther from) the global benchmark technology frontier.

### Selection of variables

#### Variables for Super-SBM DEA

Based on the definition of the healthcare services efficiency presented in this study and the existing literature on the selection of healthcare services efficiency indicators [23], in this study, the input indicators were selected from manpower, material, and capital, and the expected output indicators were selected from both activity and quality. The most frequently used variables from their respective categories were selected as input and output indicators, respectively. Based on the statistics in the systematic review, the selected input indicators included the number of healthcare personnel, the number of beds, and the proportion of healthcare expenditure in GDP. Output indicators included the number of consultations, bed occupancy rate, and healthcare revenue. According to China's strategy for the healthcare system, health institutions are tasked with providing basic medical services and with tasks related to public welfare, such as preventing infectious diseases. Therefore, this study enters the infectious disease death rate of categories A and B into the model as an undesirable output [24] (see Table 1 for details of the indicators).

**Table 1:** Selection of input, output, and environmental variables

Indicators Type	Indicators Meaning	Specific Indicator	Symbol	Unit
Input	Manpower	Number of healthcare personnel [25]	HP	per 1000 people
	Material	Number of beds [26]	Beds	per 1000 people
	Capital	The proportion of healthcare expenditure in GDP [27]	EIG	%
Output	Desirable	Number of consultations [24]	NC	person
		Bed occupancy rate [28]	BOR	%
		Healthcare revenue [29]	HR	million yuan
	Undesirable	Infectious disease death rates of category A and B [24]	DR	%
Environmental variables	Economic	GDP per capita [30]	GDP	yuan
	Population	Population density [31]	PD	people per km2
	Subsidy	The ratio of government subsidies to healthcare revenues [32]	RSR	%
	Market Structure	Percentage of regional tertiary hospitals [31]	PTH	%

#### Variables for Stage-2 SFA

Healthcare services efficiency is influenced not only by controllable factors such as inputs and outputs but also by environmental factors beyond the control of the healthcare organization. Scholars have identified the economic, professional, and technical environment as the determining variables for managed and unmanaged quality [33]. In this study, GDP per capita, population density, government subsidies as a percentage of healthcare revenue, and the percentage of regional tertiary hospitals were selected as environmental variables to be studied. The specific indicators are shown in Table 1.

### Data source

The input and output data, subsidy environment and market structure environment indicators data used above were selected from the China Health Statistical Yearbook 2010–2012, China Health and Family Planning Statistical Yearbook 2013–2017, China Health care Statistical Yearbook 2018–2022, and the statistical yearbooks of each province. In addition, data on the economic and population environments were obtained from the China Statistical Yearbook for 2009–2021. Since this study uses panel data, the value indicators for all years were converted to the 2009 price level to eliminate price effects. In addition, the division of the eastern, central, and western regions was based on the National Bureau of Statistics classification. The eastern region includes 11 provinces and municipalities directly under the central government, including Beijing, Tianjin, Hebei, Liaoning, Shanghai, Jiangsu, Zhejiang, Fujian, Shandong, Guangdong, and Hainan; the central region includes eight provinces of Shanxi, Jilin, Heilongjiang, Anhui, Jiangxi, Henan, Hubei, and Hunan; western region including Neimenggu, Guangxi, Chongqing, Sichuan, Guizhou, Yunnan, Xizang, Shanxi, Gansu, Qinghai, Ningxia, Xinjiang 12 provinces, autonomous regions and municipalities directly under the Central Government.

Matlab software was used to measure the healthcare services efficiency index and the growth of healthcare services and its decomposition for 31 provinces in China. SFA regression results are done with the help of frontier 4.1.

## Results

### Overall and provincial results of China's Health Service Efficiency Index (HSE)

The three-stage super-efficient SBM method was used to measure the mean values of HSE before and after optimization in eastern, central, western, and 31 provinces of China from 2009 to 2021; the results are shown in Table 2.

**Table 2:** HSE mean values for 2009–2021 of 31 provinces and each region in China

Area	Region	phase I DEA			phase III DEA		
		HSE	PTE^G^	SE^G^	HSE_1	PTE^G^ _1	SE^G^ _1
East	Beijing	0.266	0.273	0.971	0.729	0.767	0.961
	Tianjin	0.212	0.220	0.963	0.250	0.277	0.913
	Hebei	0.708	0.747	0.943	0.447	0.463	0.962
	Liaoning	0.319	0.333	0.955	0.283	0.308	0.920
	Shanghai	0.400	0.812	0.520	0.728	0.929	0.778
	Jiangsu	0.901	0.959	0.937	0.761	0.807	0.941
	Zhejiang	0.690	0.799	0.874	0.790	0.865	0.907
	Fujian	0.763	0.836	0.900	0.428	0.452	0.946
	Shandong	0.774	0.854	0.900	0.599	0.615	0.972
	Guangdong	0.968	0.997	0.971	0.925	0.953	0.971
	Hainan	0.099	0.109	0.916	0.115	0.128	0.905
	Mean	0.554	0.631	0.895	0.550	0.597	0.925
Central	Shanxi	0.286	0.332	0.863	0.206	0.213	0.972
	Jilin	0.189	0.202	0.936	0.197	0.207	0.953
	Heilongjiang	0.206	0.213	0.962	0.219	0.234	0.941
	Anhui	0.654	0.674	0.970	0.460	0.503	0.927
	Jiangxi	0.582	0.595	0.976	0.482	0.573	0.869
	Henan	0.683	0.705	0.969	0.799	0.844	0.946
	Hubei	0.585	0.707	0.840	0.557	0.721	0.779
	Hunan	0.454	0.486	0.940	0.507	0.541	0.941
	Mean	0.455	0.489	0.932	0.429	0.479	0.916
West	Neimenggu	0.385	0.482	0.803	0.179	0.191	0.941
	Guangxi	0.427	0.496	0.895	0.870	0.975	0.893
	Chongqing	0.400	0.456	0.898	0.408	0.431	0.951
	Sichuan	0.577	0.635	0.909	0.883	0.915	0.963
	Guizhou	0.403	0.411	0.981	0.439	0.504	0.911
	Yunnan	0.386	0.392	0.985	0.673	0.787	0.865
	Xizang	0.038	0.142	0.577	0.032	0.130	0.608
	Shaanxi	0.313	0.333	0.945	0.283	0.295	0.956
	Gansu	0.308	0.392	0.891	0.173	0.181	0.952
	Qinghai	0.071	0.085	0.868	0.049	0.053	0.932
	Ningxia	0.237	0.265	0.900	0.071	0.078	0.921
	Xinjiang	0.165	0.188	0.886	0.255	0.271	0.941
	Mean	0.309	0.356	0.878	0.360	0.401	0.903
	National mean	0.434	0.488	0.898	0.445	0.491	0.914

*Note*: HSE is the healthcare efficiency index, representing the global healthcare services comprehensive technical efficiency, PTE^G^ represents the global pure technical efficiency, and SE^G^ represents the global scale efficiency.$HSE={PTE}^{G}\times {SE}^{G}$. Accordingly, HSE_1, PTE^G^_1, and SE^G^_1 represent HSE, PTE^G^ and SE^G^ after optimization by the SFA equation respectively, and $\mathrm{HSE}\_1= {PTE}^{\mathrm{G}}\_1\times {SE}^{G}\_1$

From Table 2, it can be observed that the efficiency values differ significantly between provinces. This is because the results of this study are measured using the global SBM method, where the 31 decision units share the same production frontier for the data from 2009 to 2021. This results in the difference between the highest and lowest efficiency values being the result of 13 years of accumulation. However, the advantage of this method is that the efficiency levels of each decision unit in different time dimensions can be directly compared.

By comparing the efficiency values of the first and third stages, the optimized average HSE for 2009–2021 in China increased from 0.434 to 0.445. This is an effect caused by the presence of environmental variables and statistical noise, indicating that environmental variables and random noise have a negative impact on HSE, which means that the HSE in the first stage is underestimated. Similarly, the PTE^G^ and the SEG in the third stage was higher than before optimization, indicating that environmental variables and statistical noise weaken the technical and scale management level of healthcare services in China. Therefore, it is necessary to conduct the second-stage SFA regression to remove the effects of environmental factors and random noise. Furthermore, the decision units were all placed in the same external environment to more accurately measure the healthcare services efficiency.

**Fig. 1:**
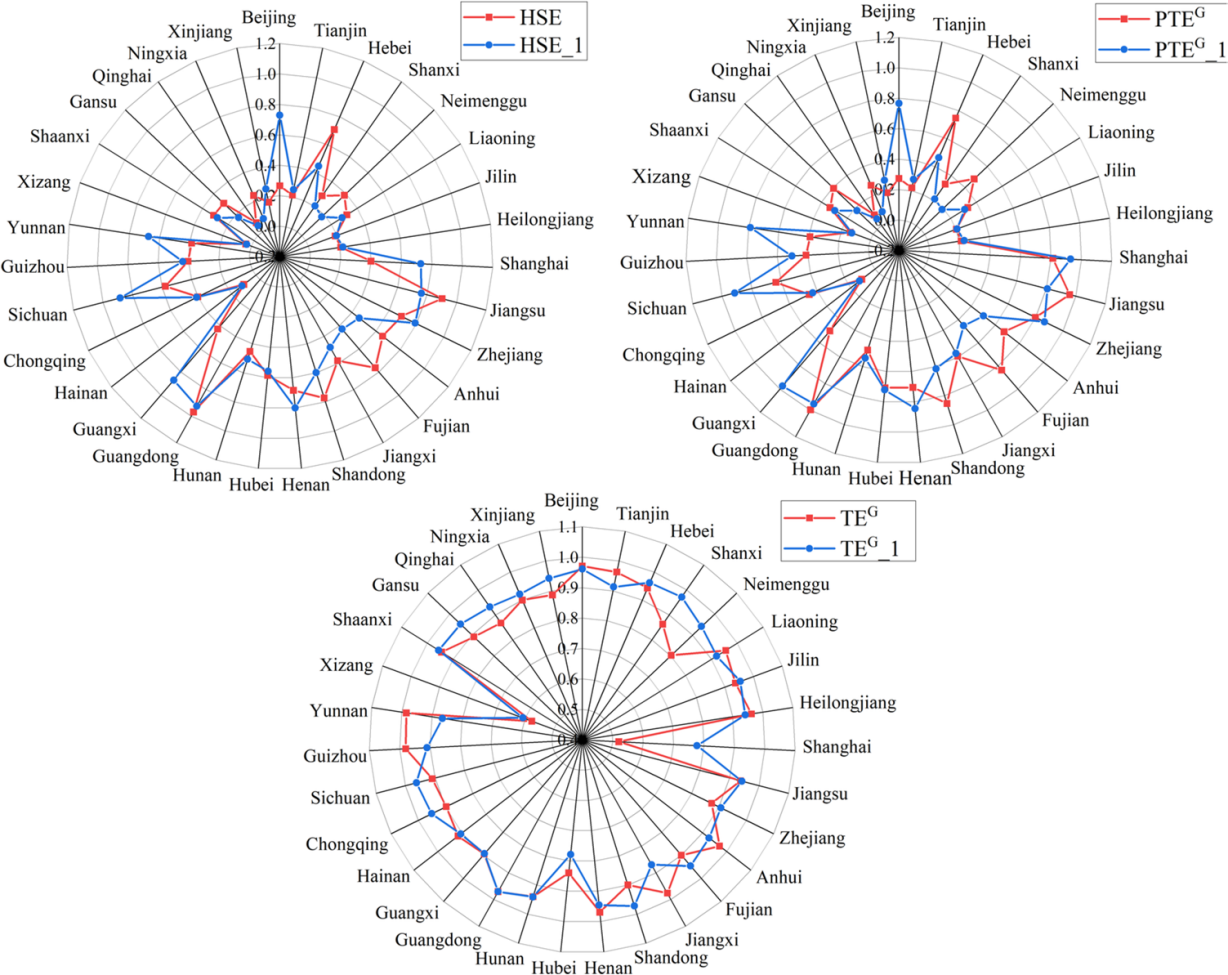
Comparison of HSE, PTE^G^ and TE^G^ in 31 provinces between the first stage and the third stage

The comparison of the efficiency values of each province before and after optimization is shown in Fig. 1. The comparison shows that the HSE and PTE^G^ of the 31 provinces have a similar pattern of change after SFA adjustment. The HSE and PTE^G^ in Beijing, Shanghai, Guangxi, Sichuan and Yunnan show a large increase after adjustment, indicating that the combination of various environmental factors in these regions plays an inverse role on the efficiency values. In the case of Beijing, its larger population density compared to other provinces may lead to an underestimation of efficiency values. In contrast, HSE and PTEG values in Hebei and Fujian are considerably lower after adjustment. This may be related to their lower economic conditions. Compared to HSE and PTEG, the difference between the efficiency values of SEG before and after adjustment is relatively small. Since HSE is the result of the product of PTE^G^ and SE^G^, the greater of the two change rates determines the direction of change of HSE. The radar plot reveals that the direction of change of HSE in most provinces is consistent with the change of PTE^G^, which is also evident from the similar change pattern of both before and after adjustment. After the second stage of SFA regression analysis, Jiangsu, and Guangdong provinces remain at the higher frontier of China's healthcare services levels, with little change in average efficiency values, while Qinghai, and Xizang remained at the bottom of the adjusted HSE. This is in line with our usual perception that the eastern region has better development conditions and opportunities compared to the western region.

Table 3 shows the development of the healthcare services efficiency in each province during the study period. Taking Beijing as an example, HSE showed a stable upward trend from 0.182 in 2009 to more than 1 in 2019, reaching a maximum value of 1.029 in 2021. It is worth noting that almost all Chinese provinces experienced some degree of decline in HSE in 2013, with only Beijing, and Shanghai growing in reverse. This phenomenon is attributed to the government's shift in focus of healthcare reform in 2012, which retarded the growth of healthcare services efficiency, and Beijing and Shanghai have resisted the decline in healthcare services efficiency with their ability to manage risk. In addition, Jiangsu, Guangdong, Sichuan, Henan, Zhejiang, Guangxi, Beijing and Shanghai showed an average HSE > 0.7 at the top level nationwide. Hubei, Shandong, Hunan, Yunnan, Fujian, Jiangxi, Hebei, Anhui, Guizhou and Chongqing showed an HSE between 0.4and 0.7. However, the HSE of Liaoning, Shaanxi, Xinjiang, Heilongjiang, Shanxi, Tianjin, Jilin, Neimenggu, Gansu, Hainan, Ningxia, Qinghai and Xizang was below 0.4 and at a lower level. It can be found that the efficiency values of healthcare services in each province of China basically match the level of local economic development, with the southeastern coastal region having higher efficiency in healthcare services, the central region having the second highest, and the western and northeastern regions having the lowest efficiency values.

**Table 3:** Annual HSE mean values of 31 provinces(municipalities) in China

year	2009	2010	2011	2012	2013	2014	2015	2016	2017	2018	2019	2020	2021	Mean value
province														
Beijing	0.182	0.260	0.436	0.641	1.000	0.696	0.769	0.842	0.858	0.966	1.020	0.773	1.029	0.729
Tianjin	0.092	0.141	0.196	0.276	0.233	0.280	0.272	0.280	0.308	0.283	0.316	0.263	0.304	0.250
Hebei	0.233	0.422	0.439	0.463	0.280	0.490	0.482	0.510	0.496	0.500	0.515	0.489	0.492	0.447
Shanxi	0.099	0.144	0.175	0.207	0.128	0.223	0.227	0.234	0.244	0.246	0.256	0.240	0.260	0.206
Neimenggu	0.087	0.160	0.164	0.186	0.118	0.190	0.196	0.207	0.210	0.211	0.210	0.197	0.197	0.179
Liaoning	0.156	0.224	0.250	0.276	0.210	0.295	0.296	0.302	0.312	0.357	0.367	0.308	0.325	0.283
Jilin	0.096	0.142	0.158	0.180	0.123	0.196	0.221	0.228	0.243	0.241	0.255	0.231	0.249	0.197
Heilongjiang	0.127	0.185	0.204	0.217	0.166	0.224	0.234	0.248	0.279	0.288	0.284	0.194	0.201	0.219
Shanghai	0.246	0.337	0.528	0.587	1.014	0.656	0.599	0.768	0.836	0.958	1.002	0.890	1.036	0.728
Jiangsu	0.525	0.743	0.749	0.842	0.633	0.865	0.828	0.873	0.763	0.769	0.780	0.825	0.693	0.761
Zhejiang	0.409	0.581	0.719	0.785	0.622	0.842	0.806	0.858	0.862	0.885	1.013	0.881	1.007	0.790
Anhui	0.380	0.704	0.534	0.456	0.258	0.463	0.433	0.459	0.460	0.444	0.480	0.448	0.460	0.460
Fujian	0.341	0.437	0.394	0.405	0.246	0.414	0.404	0.424	0.465	0.505	0.517	0.484	0.526	0.428
Jiangxi	0.386	0.606	1.004	0.460	0.203	0.409	0.419	0.454	0.407	0.428	0.468	0.493	0.531	0.482
Shandong	0.342	0.576	0.619	0.604	0.400	0.613	0.687	0.711	0.688	0.651	0.627	0.654	0.616	0.599
Henan	0.475	0.745	0.768	0.734	0.488	0.819	0.829	1.001	0.867	0.887	0.910	0.899	0.969	0.799
Hubei	0.377	0.632	0.734	0.557	0.294	0.487	0.474	0.494	0.514	0.542	0.576	1.008	0.549	0.557
Hunan	0.301	0.579	0.600	0.531	0.301	0.534	0.462	0.535	0.566	0.558	0.576	0.517	0.536	0.507
Guangdong	0.609	0.929	0.845	1.040	0.757	1.005	0.938	0.959	0.933	1.001	1.019	0.958	1.026	0.925
Guangxi	0.470	0.840	1.016	1.019	1.010	0.950	0.887	0.865	0.837	0.851	1.025	0.807	0.728	0.870
Hainan	0.059	0.163	0.134	0.113	0.057	0.098	0.097	0.101	0.193	0.114	0.118	0.124	0.117	0.115
Chongqing	0.219	0.394	0.423	0.392	0.228	0.382	0.385	0.417	0.416	0.446	0.501	0.526	0.580	0.408
Sichuan	0.569	0.816	1.002	0.847	0.575	0.879	0.883	0.916	0.922	1.024	1.024	1.002	1.016	0.883
Guizhou	1.001	0.543	0.468	0.344	0.182	0.311	0.324	0.340	0.380	0.410	0.469	0.470	0.466	0.439
Yunnan	0.439	0.696	0.724	0.719	0.461	0.687	0.689	0.722	0.750	0.751	0.725	0.679	0.705	0.673
Xizang	0.016	0.022	0.028	0.040	0.016	0.033	0.028	0.033	0.033	0.035	0.040	0.044	0.046	0.032
Shaanxi	0.137	0.247	0.254	0.283	0.184	0.285	0.292	0.303	0.313	0.341	0.368	0.324	0.346	0.283
Gansu	0.094	0.161	0.176	0.168	0.100	0.195	0.190	0.196	0.200	0.194	0.193	0.186	0.192	0.173
Qinghai	0.024	0.042	0.040	0.046	0.030	0.046	0.050	0.051	0.055	0.063	0.060	0.063	0.067	0.049
Ningxia	0.036	0.066	0.066	0.069	0.046	0.070	0.072	0.080	0.079	0.084	0.089	0.084	0.088	0.071
Xinjiang	0.094	0.171	0.190	0.207	0.164	0.244	0.270	0.289	0.288	0.305	0.373	0.343	0.371	0.255

### Annual HSE mean values in various regions of China

**Fig. 2:**
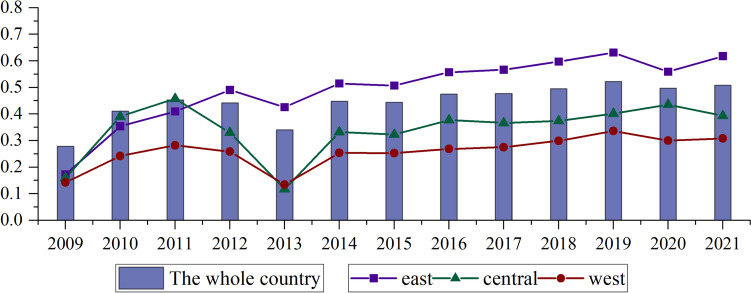
Variation trends in adjusted annual HSE averages of the whole country and each region

Each region in China was analyzed from the time dimension. After using SFA regression analysis to remove the environmental factors and random noise from the DEA model in the first stage, the trends of HSE for China as a whole, east, central and west from 2009–2021 were obtained in the third stage. The results are shown in Fig. 2. As shown in the figure, the HSE went from high to low in the order of eastern, central and western regions, respectively. The HSE in the eastern region is higher than the national average, the HSE in the western region is lower than the national average, and the central region remains in line with the national average. The ranking of health service levels in the three regions corresponds to the regional economic development status. This may be due to the fact that provinces with higher levels of economic development have advanced medical technology and a full range of large medical equipment, which can ensure higher input–output efficiency. The national HSE increased from 0.278 in 2009 to 0.445 in 2021 (an increase of 82.45%). The HSE for the Eastern, Central and Western regions increased from 0.290, 0.280 and 0.265 in 2009 to 0.550, 0.429 and 0.360 in 2021, representing increases of 124.46%, 67.5% and 50.82%, respectively.

From the changing trend, the change in HSE was almost consistent across regions. It showed an increasing trend and a gradual slowdown from 2009 to 2012 and then significantly dropped in 2013, and remained relatively stable after 2014.. This phenomenon is related to the government’s reorientation of healthcare reform priorities in 2012, as analyzed above. Another point worth noting is that the efficiency values of healthcare services in all three regions and the country as a whole decline to some extent in 2020 and 2021. This is because the outbreak of COVID-19 in China at the end of 2019 severely depleted healthcare resources and occupied a large number of healthcare workers. This highlights the shortcomings of the lack of healthcare resources in China's public health sector.

**Fig. 3:**
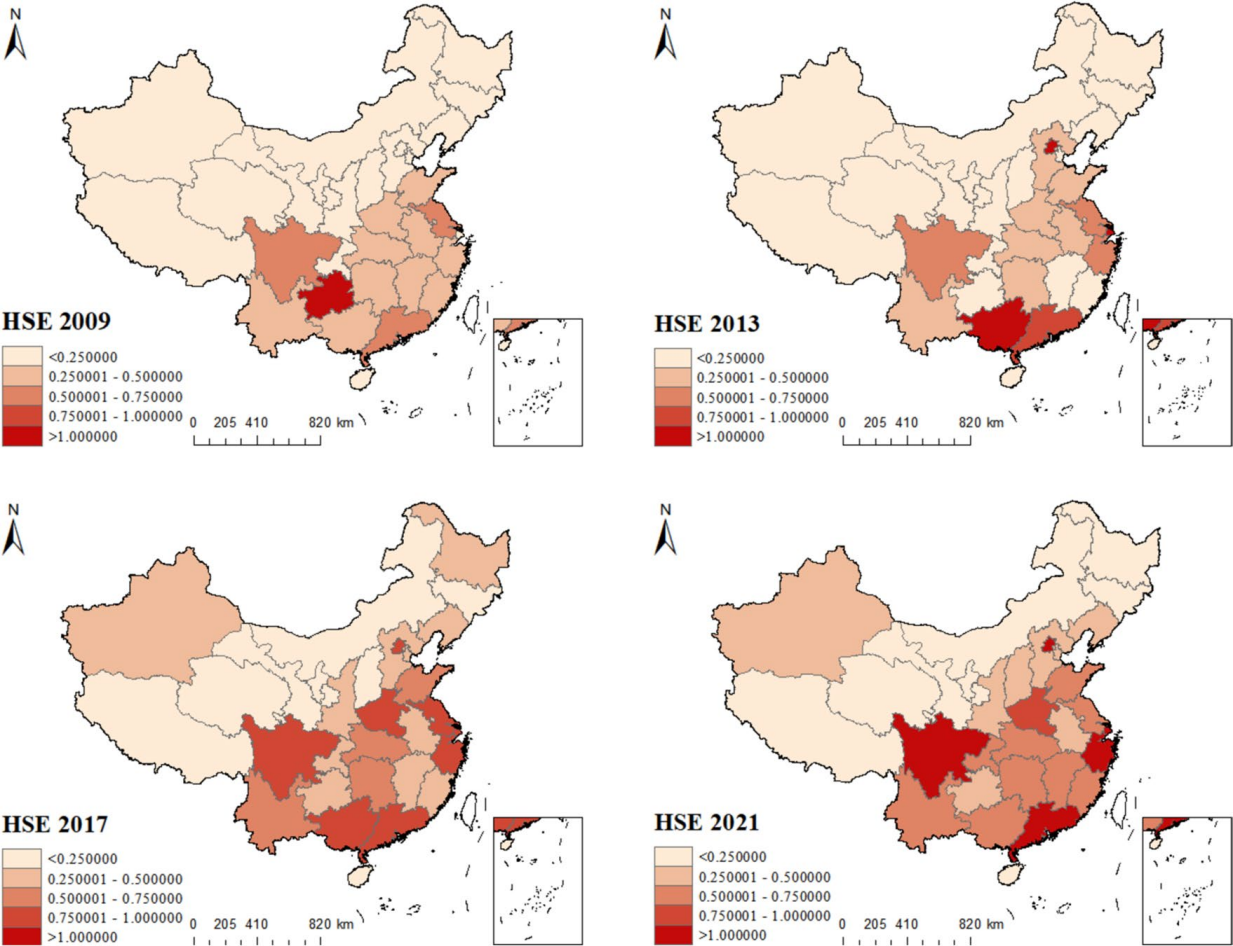
The evolution of HSE patterns of 31 provinces in China

ArcGIS 10.2 is used to display the HSE of 31 provinces in China from 2009–2021 to help get a more accurate picture of the development of China's healthcare services efficiency. Figure 3 shows the HSE values for 31 Chinese provinces in 2009, 2013, 2017, and 2021, and the trend of HSE evolution in China during the study period. The shades of regional colors represent the magnitude of HSE, with darker colors indicating larger HSE values. From the evolution trend, the most intuitive expression is that the number of light red areas (HSE < 0.25) gradually decreases, and the number of dark red areas (HSE > 1) gradually increases; the area color gradually deepens over time. It indicates that the HSE of most provinces gradually increased over time. In particular, the number of regions with HSE > 0.75 increased significantly for the data in 2017.

It can be found that during the period after the healthcare reform, the provinces with greater efficiency growth in healthcare services are mainly located in the southeastern coastal region, while the efficiency values of almost all provinces in the western and northeastern regions consistently remain below 0.5. This reflects the many problems in the allocation of China's healthcare resources. As we all know, China has a vast territory with varying levels of regional development, and healthcare resources and high-level medical talents are mainly distributed in the economically developed southeastern regions, while the central and western regions are disadvantaged by geography and other disadvantages, resulting in resource scarcity problems. Local governments are also making continuous efforts to promote equity in the allocation of health resources, and Henan's efficiency growth is due to the government's policy support for the medical field in the central region. Similarly, the overall level of healthcare services in the western region has increased, but the problem of uneven resource allocation within the region remains prominent. As indicated in Fig. 3, medical resources in the western region are concentrated in Sichuan Province, while Qinghai, Tibet, and Ningxia remain scarce.

### Analysis of the external environment for the HSE in China

Based on the SFA regression model, this study explored the effects of external environmental variables on the slack in the three input variables. Calculations were performed according to Eq. (3), and the regression results are shown in Table 4. The likelihood ratio test shows that each regression is significant at the 1% significance level, indicating that applying the SFA model is reasonable.

**Table 4:** SFA regression model results

Environmental variable	Number of healthcare personnel	Number of beds	The proportion of healthcare expenditure in GDP
Constant	-2.2527 (0.3174)	-1.0104 (0.1876)	-0.5035 (0.2526)
Economic	0.0001*** (0.0001)	0.0001*** (0.0001)	-0.0001 (0.0001)
Population	0.0004*** (0.0001)	-0.0002** (0.0001)	-0.0001 (0.0002)
Subsidies	0.0448*** (0.0144)	0.0229*** (0.0068)	0.0448*** (0.0106)
Technology	0.0132 (0.0100)	0.0002 (0.0060)	0.0051 (0.0103)
Sigma-squared	9.2159	12.2605	7.3902
Gamma	0.9155	0.9793	0.8952
Log-likelihood function	-564.3265	-356.7315	-555.9519
LR test	196.5064	579.8620	217.8550

^***^,**,* indicate t-values significant at the 1%, 5%, and 10% significance levels, respectively. The number in parentheses indicates the standard deviation

As shown in Table 4, the effects of environmental variables in terms of economy, population, and government subsidies on input redundancy passed the t-test, and only the population and economy environment had a non-significant effect on capital input redundancy in the healthcare system. This indicated a valid relationship between these external environments and input redundancy in healthcare services. The technology environment variables were insignificant, indicating that the percentage of regional tertiary hospitals did not affect the input redundancy of healthcare services. The Gamma values of the input redundancy regression models were all close to 1, indicating that external environment management noise was the key factor dominating the variation of China's healthcare services efficiency. The effect of management ineffectiveness was greater than random error, and the management capacity of Chinese healthcare services was slightly weaker.

In terms of the economic environment, it positively affects the slack in all input variables of healthcare services. This suggests that a positive economic environment instead increases resource redundancy in Chinese healthcare services, resulting in redundancy spillover of input factors. The reason for this may be that the better the economic environment, the more healthcare providers invest in manpower, equipment and capital, resulting in an increase in the scale beyond the absorptive capacity of society, resulting in resource redundancy. In economically advantaged areas, when too many input resources are not fully utilized, it wastes resources and is not conducive to the efficiency of medical services.

In terms of the demographic environment, population density has a positive effect on the redundancy of manpower resource inputs, suggesting that healthcare systems in provinces with higher population density have a problem of over-enrolling healthcare personnels. However, population density has a significant negative effect on the redundancy of the number of beds, suggesting that increased population density leads to less redundancy in healthcare bed inputs, which in turn improves healthcare services efficiency. Higher population density indicates that healthcare institutions are catering to a larger market and more potential patients. The increase in patients will eliminate the redundancy of healthcare bed inputs and even trigger a shortage of beds. When the input elements of each unit are fully utilized, there are no resource constraints or waste, ensuring improved healthcare services efficiency.

In terms of the subsidy environment, government subsidies have a significant positive effect on input redundancy in healthcare personnel, number of beds, and healthcare expenditures, suggesting that an increase in government subsidies leads to an increase in healthcare input redundancy, which in turn reduces healthcare services efficiency. This may be explained by the fact that Chinese government subsidies to the healthcare system may impact the opposite direction. The fact that the Chinese government's per capita subsidies to the healthcare system do not directly fund healthcare services may be the source of the problem. Therefore, the Chinese government needs to rationalize its subsidy program in the context of reality in order to improve healthcare services efficiency.

### National and regional HSGI change and its decomposition

To analyze the intrinsic drivers of HSGI in China, a global Malmquist-Luenberger production index and its decomposition technique proposed in the previous section were used to explore the growth of health services efficiency in terms of the “catch-up effect” of provinces on the current benchmark technology frontier and the “innovation effect” of healthcare services efficiency growth in each province.

**Fig. 4:**
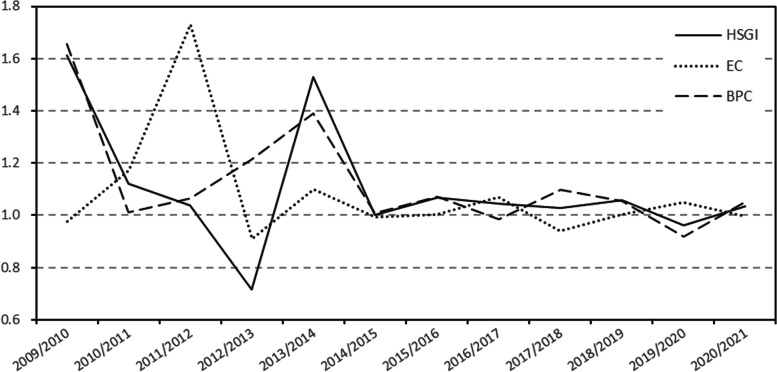
TFP changes and the decomposition of HSGI in China, 2009–2021

The changes in HSGI and its decomposition are shown in Fig. 4. From 2009 to 2012, HSGI was greater than 1 and then gradually decreased. This indicates that the efficiency of healthcare services in China increased significantly during that period, and the growth rate gradually slowed with time. There was a trough of HSGI in 2013, which was 31.1% lower than the previous year. In 2014, HSE increased again by 113.7% compared to the previous year, and healthcare services efficiency returned to the same level as in 2012. During the subsequent study period, the HSGI was maintained near 1, indicating that the efficiency of healthcare services remained near the level value of 2014 with less fluctuation.

To explain the change in healthcare services efficiency, the decomposition of HSGI allows the identification of the change in technical efficiency (EC) and the change in the healthcare services efficiency gap between the current technical frontier and the global frontier (BPC). The fluctuation of EC tends to slow down over time, with the final value remaining around 1 and tending to be greater than 1. These data indicates that the level of healthcare services efficiency in China has improved, and the catch-up effect has gradually remained stable.

The BPC trend is consistent with HSGI, indicating that BPC mainly contributes to the change of HSGI, and the efficiency change of Chinese healthcare services mainly depends on the innovation effect. In other words, the Chinese healthcare services is currently relying on technological innovation and progress to move closer to the global technology frontier. BPC experienced large fluctuations from 2009–2014 but was always greater than 1, indicating that the innovation effect of Chinese healthcare services has always played a positive role in improving efficiency. After 2014, the BPC fluctuated around 1, but the effect was insignificant, indicating that the innovation effect of Chinese healthcare services remains relatively stable.

The changes in the efficiency of healthcare services by region and the decomposition of HSGI are shown in Table 5. Based on economic development and geographic location, China can be divided into three regions, i.e., eastern, central, and western area. As shown in Table 5, the average HSGI for the eastern region was 1.108 during the study period, with an average annual growth rate of 10.8% for the healthcare services efficiency. The eastern region had an EC of 1.033 with an average annual growth rate of 3.3% and a BPC of 9.1%. The central region had an average HSGI of 1.095 with an average annual growth rate of 9.5%. The average annual growth rates of EC was 2.7%, while average annual decrease of BPC is 0.5%. The western region led these three regions with an HSGI of 1.101 and an average annual growth rate of 10.1%. EC had an average annual growth rate of 16.9%. With an average annual BPC growth rate of 19.5%, the Western region contributed the most of the three regions. These results indicate that the growth of the healthcare services efficiency in these three regions is facilitated by both the catch-up effect and the innovation effect, but the innovation effect is the most important driver, and the gap between the current technology frontier and the global technology frontier is gradually narrowing. This implies that the current Chinese healthcare services system relies on technological innovation and progress and is moving closer to the technology frontier rather than relying solely on increased investment in health resources. The HSGI and its two decomposition items showed an increase or decrease in fluctuations. In 2013, the HSGI and BPC values for all three regions were at a low point, which is consistent with the previous analysis of the HSE. The reason for this may be the negative impact of the 2012 refocusing of the healthcare system reform, which affected the “innovation effect” in the development of healthcare services in China, thus affecting the growth of healthcare services efficiency.

**Table 5:** The change and decomposition of HSGI for each region in China

Time	National			Eastern region			Central region			Western region		
	HSGI	EC	BPC	HSGI	EC	BPC	HSGI	EC	BPC	HSGI	EC	BPC
2009/2010	1.612	0.977	1.656	1.605	0.953	1.685	1.621	1.000	1.632	1.613	0.983	1.645
2010/2011	1.120	1.171	1.012	1.159	1.435	0.873	1.134	1.039	1.104	1.074	1.017	1.078
2011/2012	1.038	1.732	1.066	1.131	0.936	1.204	0.913	0.904	1.018	1.036	3.013	0.971
2012/2013	0.715	0.909	1.214	0.867	0.970	0.881	0.604	0.857	0.713	0.650	0.887	1.853
2013/2014	1.529	1.100	1.390	1.336	1.050	1.265	1.700	1.221	1.413	1.591	1.065	1.491
2014/2015	1.000	0.993	1.008	0.992	0.988	1.006	1.000	0.979	1.023	1.008	1.007	1.001
2015/2016	1.067	1.003	1.070	1.068	1.012	1.056	1.084	0.976	1.129	1.055	1.012	1.043
2016/2017	1.045	1.071	0.984	1.094	1.136	0.974	1.012	1.021	1.001	1.075	1.005	1.070
2017/2018	1.028	0.940	1.099	1.007	0.924	1.097	1.014	0.908	1.117	1.055	0.964	1.173
2018/2019	1.057	1.004	1.053	1.043	1.011	1.034	1.048	0.995	1.053	1.057	0.975	1.089
2019/2020	0.962	1.050	0.917	0.923	1.024	0.905	1.018	1.098	0.923	0.961	1.042	0.925
2020/2021	1.035	0.995	1.051	1.068	0.959	1.114	0.995	0.947	1.047	1.033	1.061	0.997
Mean	1.101	1.079	1.127	1.108	1.033	1.091	1.095	0.995	1.098	1.101	1.169	1.195

Comparing the healthcare services efficiency of each region in Table 2 reveals that although the western region has the lowest HSE among all regions, its EC and BPC values are higher than all other regions. Also, the BPC of the western region dominates the national healthcare services efficiency growth, which may be closely related to the state’s support policies. After the launch of the healthcare system reform, the state has increased its investment in healthcare in the western region. Western provinces and cities are also making efforts to build the infrastructure of medical institutions, and the healthcare services system has been improving.

## Discussion

In order to more reasonably and accurately assess healthcare services efficiency, we constructed a three-stage super-efficient SBM model and considered the undesired outputs in the operation of the healthcare system to measure the healthcare services efficiency and its growth of 31 provinces in China from 2009–2021. The HSE and ranking of each region changed after eliminating the effects of environmental factors and statistical noise with the help of the SFA equation. These results indicate that it is necessary to place each decision unit under the same luck to obtain robust results when evaluating healthcare services efficiency in China. After eliminating the effects of environmental factors and statistical noise, China's average healthcare services efficiency improved from 0.434 to 0.445. In addition, the efficiency values showed an inverted u-shaped trend from 2009 to 2013, followed by a slow upward trend. On average, the level of healthcare services efficiency in the eastern region was higher than that in the central and western regions (being the lowest in the western region), which may be related to the imbalance in the national allocation of healthcare resources among regions. These data are consistent with Liu et al. [2], who found better healthcare resources in the eastern region.

Most of the environmental variables used in this analysis significantly affect the redundancy of health resource inputs. For example, we found that an increase in population density reduces the redundancy of material input resources, which is consistent with Bates et al. [34] and Yousefi Nayer et al. [35]. However, this study found that in China, provinces with high population density have more redundancy in manpower input resources. Moreover, economic development as well as increased government subsidies can cause redundancy in healthcare resources. Similarly, Chen et al. [24] obtained the same results by assessing Chinese public hospitals. Therefore, the Chinese government has to adjust its healthcare system reform strategy and cannot rely solely on increased resource input to try to improve the efficiency of healthcare services, which may have the opposite result and burden the healthcare system.

The present study also found that the percentage of regional tertiary hospitals did not significantly affect each input variable, suggesting that the expansion of hospital size does not necessarily improve the efficiency of healthcare services. This differs from the results obtained in previous studies, probably because this paper considers non-desired outputs that are not considered in most of the literature and eliminates both environmental variables and statistical noise from interfering with the assessment of healthcare services efficiency. There is a consensus that expanding the size of a hospital makes better use of the available medical talent, infrastructure, and equipment. However, Giancotti et al. [36] found that the relationship between healthcare services efficiency and hospital size has a "concave curve" and that a hospital size above or below the "appropriate size" would result in a decrease in healthcare effectiveness.

The healthcare services efficiency assessment results found large differences in the values of HSE in the three regions of China. It may be that several regions in China have different socio-economic and different roles in the planning of the national health system, and there are differences in the demand and supply of health services, which lead to differences in operational efficiency and total factor productivity. Nevertheless, the study found that the HSE of the national and three regions showed similar trends of increase and decrease over the study period, with a "sharp increase" from 2009 to 2012. This is because the government increased its investment in healthcare resources in the pre-reform period. However, this rapid growth due to the reform policy was not sustainable, which is consistent with other scholars' findings [37]. In 2012, the government adjusted the focus of healthcare reform, and the growth of healthcare services efficiency slowed down, and in 2013, the national healthcare services efficiency declined for the first time. Except for a decline in 2013, all other years showed varying degrees of increase. This indicates that China's healthcare services efficiency has improved since the healthcare reform compared to the previous years.

From the perspective of China's healthcare services efficiency growth, most of the HSGI were above 1 in three regions during the study period. In particular, the western region showed an average annual growth of 10.1% in the healthcare services efficiency index from 2009 to 2021 since the implementation of healthcare system reform. This suggests that the strong policy support provided by the healthcare reform and the basic medical hardware have improved China's healthcare services efficiency, which is particularly obvious in the western region. Therefore, it can be said that the new round of healthcare system reform is more applicable to the western region. The decomposition of the HSGI shows that the BPC mainly contributes to the increase in China's healthcare services efficiency, indicating that China's healthcare reform does not only rely on increasing the investment of healthcare resources to improve the healthcare services efficiency but also promotes the technological innovation of the healthcare system, which is consistent with results reported by Gong et al. [38].

## Conclusion

In this paper, a three-stage super-efficiency SBM model was used to assess the efficiency of healthcare services in China after the healthcare system reform, and the HSGI index and its decomposition were used to analyze the intrinsic influence mechanism of healthcare efficiency efficiency growth. The conclusions based on the analysis are summarized as follows: the average level of healthcare services efficiency in 31 provinces of China after the healthcare system reform showed a fluctuating upward trend, and there were regional differences in the efficiency of healthcare services in China. Environmental factors had a significant impact on the efficiency of healthcare services in China, and the increase in population density helped to reduce resource redundancy. Due to unreasonable incentive mechanism of the healthcare system, economic condition and the growth of government healthcare expenditure had a negative impact on the efficiency of healthcare services. The decomposition results of HSGI showed that the catch-up effect between provinces in China was not significant, and was mainly the innovation effect that contributed to the growth of healthcare services efficiency.

To this end, the following recommendations have been proposed: first, the government should rationalize allocating health resources according to the regional demographic and actual medical needs and appropriately develop the primary healthcare system. Primary healthcare is considered an effective health service [39], but the distribution of healthcare resources in China is seriously unbalanced between hospitals and primary healthcare institutions, resulting in unnecessary waste of healthcare resources. Second, the Chinese government should change the way of subsidy. As an important regulatory tool, public subsidies should serve as an incentive to induce better efficiency in the healthcare system. However, there is currently a moral hazard that presents a negative effect of subsidies. Therefore, the Chinese government should subsidize healthcare institutions based on actual healthcare services rather than per capita. Third, it is necessary to control the size of large hospitals. Restricting the blind expansion of large tertiary hospitals and controlling the size of large tertiary hospitals are important measures to improve the efficiency of healthcare services and reduce medical costs, as well as a necessary means to control unnecessary waste of medical resources. Fourth, the relevant departments worldwide should combine local socio-economic characteristics to give full play to their respective advantages, strengthen technological innovation in medical services, fully mobilize the catch-up effect of China's medical services and drive the common development of healthcare services in the surrounding areas.

This study refines the existing literature. First, mortality rates from infectious diseases A and B were considered as a non-desired output to be included in the healthcare services efficiency calculation. Second, a super-efficient SBM model was used to differentiate the efficient subjects, and SFA regression analysis considered both environmental factors and statistical noise to ensure the most accurate measurement of healthcare services efficiency. Third, we analyzed the intrinsic drivers of healthcare efficiency growth in China from the perspectives of "catch-up effect" and "innovation effect". The limitation of this paper is that each province is considered as a whole, and the health services productivity index is analyzed in terms of non-expected output, but this simple treatment actually leads to some errors. In addition, different provinces may pursue different purposes due to their geographical differences. Therefore, future studies can introduce regional differences into the model to get more accurate results.

## Data Availability

The datasets generated and analysed during the current study are available in the China Health Statistics Yearbook (http://www.nhc.gov.cn/mohwsbwstjxxzx/tjzxtjsj/tjsj_list.shtml) and the China Statistical Yearbook (http://www.stats.gov.cn/sj/ndsj) repository.

## Abbreviations

DEA: Data envelopment analysis
GML: Global Malmquist-Luenberger
TFP: Total factor productivity
SFA: Stochastic frontier analysis
HSE: Healthcare services efficiency index
HSGI: Healthcare services efficiency growth index
CRS: Constant returns to scale
VRS: Variable returns to scale
TE^G^: Global comprehensive technical efficiency
PTE^G^: Global pure technical efficiency
SE^G^: Global scale efficiency
ML: Malmquist-Luenberger
TE: Technical efficiency changes
TC: Technical changes
EC: Efficiency change
BPG: Best Practice Gap
BPC: Best Practice Change
